# Supplementary material for: Just add sugar for carbohydrate induced self-assembly of curcumin
Source: Nat Commun. 2019 Feb 4;10:582. doi: 10.1038/s41467-019-08402-y (PMC6362107; doi:10.1038/s41467-019-08402-y)
Supplement: Supplementary file 1 — Supplementary Information [file 41467_2019_8402_MOESM1_ESM.pdf]

Supplementary Information

Just add sugar – carbohydrate induced self-assembly of curcumin

Sandy Wong,# Jiacheng Zhao,# Cheng Cao, Chin Ken Wong, Rhiannon P. Kuchel, Sergio De Luca, James M. Hook, Christopher J. Garvey, Sean Smith, Junming Ho, Martina H. Stenzel

Supplementary Methods

Materials

D-fructose (99%, Aldrich), curcumin (>75%, Aldrich), Dopamine hydrochloride (98%, Aldrich), Tris base (>99.9%, Aldrich) were used as received.

Analytical techniques

UV-Vis spectroscopy

The UV-Vis spectra of curcumin/fructose solution were measured with Cary 300 UV–vis spectrophotometer. Fructose (100 mg) was dissolved in milliQ water (2 mL) and curcumin/DMSO solution (8 µL ,2.5 mg/mL) was added dropwise with gentle stirring for a starting curcumin (10 µg/mL) and fructose (50 mg/mL) solution. The samples were placed in the 1 cm path length quartz cuvette and the absorption was measured from 200 to 800 nm at room temperature at each fructose concentrations of 120, 170 and 200 mg/mL for each interval measurement.

Fluorescence spectroscopy

The fluorescence spectra of curcumin/fructose solutions were measured with Cary Eclipse spectrophotometer equipped with a xenon flash lamp. Fructose (100 mg) was dissolved in milliQ water (2 mL) and curcumin/DMSO solution (8 µL, 2.5 mg/mL) was added dropwise with gentle stirring for a starting curcumin (10 µg/mL) and fructose (50 mg/mL) solution. The sample was placed in a 1 cm path length, four-sided quartz cuvette and the fluorescence spectra recorded between 450 to 800 nm at λ_{ex} = 429 nm with excitation and emission slit widths of 10 mm at room temperature. This process was repeated with fructose concentrations of 120, 170 and 200 mg/mL for each interval measurement. Different temperature ranges were obtained using a Bosch heat gun (2300W) before measurement.

Nuclear magnetic resonance (NMR)

NMR spectra were acquired on a Bruker Advance III (600 MHz) spectrometer fitted with a triple resonance cryoprobe, using D_2O and DMSO-d_6 as the solvent. All chemical shifts are stated in ppm (δ) relative to DMSO-d_6 (δ =2.50 ppm)

Small-angle X-ray scattering (SAXS)

SAXS experiments were carried at the Australian Synchrotron on the small/wide-angle X-ray scattering beamline[1] using x-rays with a wavelength, λ, of 1.127 Å. Isotropic scattering patterns were collected on a Pilatus 1 M detector with an active area of 981 x 1043 pixels of 170 m^2 each with a 2.7 m sample to detector distance. The magnitude of scattering vector (q) is defined by

$$q = \frac{4\pi}{\lambda} \sin(\frac{\theta}{2})$$

where θ is the scattering angle. The samples were placed in a 96-well plate solution autoloader from where samples were taken automatically and the SAXS measured in a consistent position in a quartz capillary. A background subtraction was performed using a solvent filled capillary scaled by the

transmission of sample and solvent capillaries. The SAXS data reduction: conversion of raw pixel counts, measurement geometry and x-ray wavelength into a radial average; background subtraction; and the normalisation to the scattered intensity of water as a secondary intensity standard were performed by the beamline program Scatterbrain.

For analysis, the Porod law was applied. The Porod law[2] is the limiting scattering law that applies to the high q-region. For the two-phase systems, the limiting of intensity of scattering is clearly related to the surface area through a limiting power law slope of -4. In the high q region, the Porod law is applied for the ideal two phase system bounded by the clear interface of area S, scattering vector (q):

$$I(q) \rightarrow \frac{2\pi(\Delta\delta)^2 S}{q^4} \qquad \text{equation 1}$$

where $(\Delta\delta)^2$is the contrast term which is analogous to refractive index differences for light. However, this law can be used in systems with the defined interfaces.

Transmission electron microscopy (TEM)

The TEM micrographs were obtained using a JEOL1400 transmission electron microscope comprising of a dispersive X-ray analyser and a Gatan CCD facilitating the acquisition of digital images. The measurement was conducted at an accelerating voltage of 80 kV. The samples were prepared by casting the micellar solution onto a copper grid. For the measurement of fructose-curcumin hollow particles, the grids were dried by air and then negatively stained with uranyl acetate for 5 minutes. No staining was conducted for the measurement of PDA-Fru-CCM hollow nanoparticles.

Cryo-transmission electron microscopy (cryo-TEM)

Cryo-TEM analyses were performed on an FEI Tecnai G2 20 TEM operating at an accelerating voltage of 200 kV. Images were acquired using a BM Eagle 2K CCD Camera and in-built low-dose software. Samples were vitrified using a Leica EM GP vitrification robot following a general procedure as follows: 4 µL of sample ([Fru]= 10 mg/mL, [CCM]= 60 µg/mL) was pipetted onto a glow discharged 300 mesh copper grid with lacey formvar/carbon film support, and allowed to equilibrate for 30 s at room temperature and a relative humidity 99%. The sample droplet was then blotted directly from the front (1.0 s blot time; 1 s drain time) and plunged into liquid ethane held at -174 °C. The vitrified grid was stored in a cryo-grid box immersed in liquid nitrogen before finally being cryo-transferred into the microscope using a Gatan 626 cryo-transfer holder.

Scanning electron microscopy (SEM)

SEM was used for observation of nanoparticle surface morphologies. To obtain SEM images, a drop of sample solution was spread on a mica plate and left to dry overnight followed by coating with platinum (30 nm) and viewed by using a NANOSEM 230 field-emission scanning electron microscope (FE-SEM) operated at 5kV with grid voltage of 250V. The images were recorded by a Everhardt-Thornley detector.

Dynamic light scattering (DLS)

The hydrodynamic diameter D_h was determined using a Malvern Zetasizer particle size analyser (laser, angle = 173°). The ζ potential determinations were based on electrophoretic mobility of the nanoparticles in the aqueous medium, which was performed using folded capillary cells in automatic mode. Different pH values were obtained and controlled by an autotitrator MPT-2 that utilized HCl acid (0.025 M) and NaOH base (0.025 M) additives with stirring to reach the desired pH.

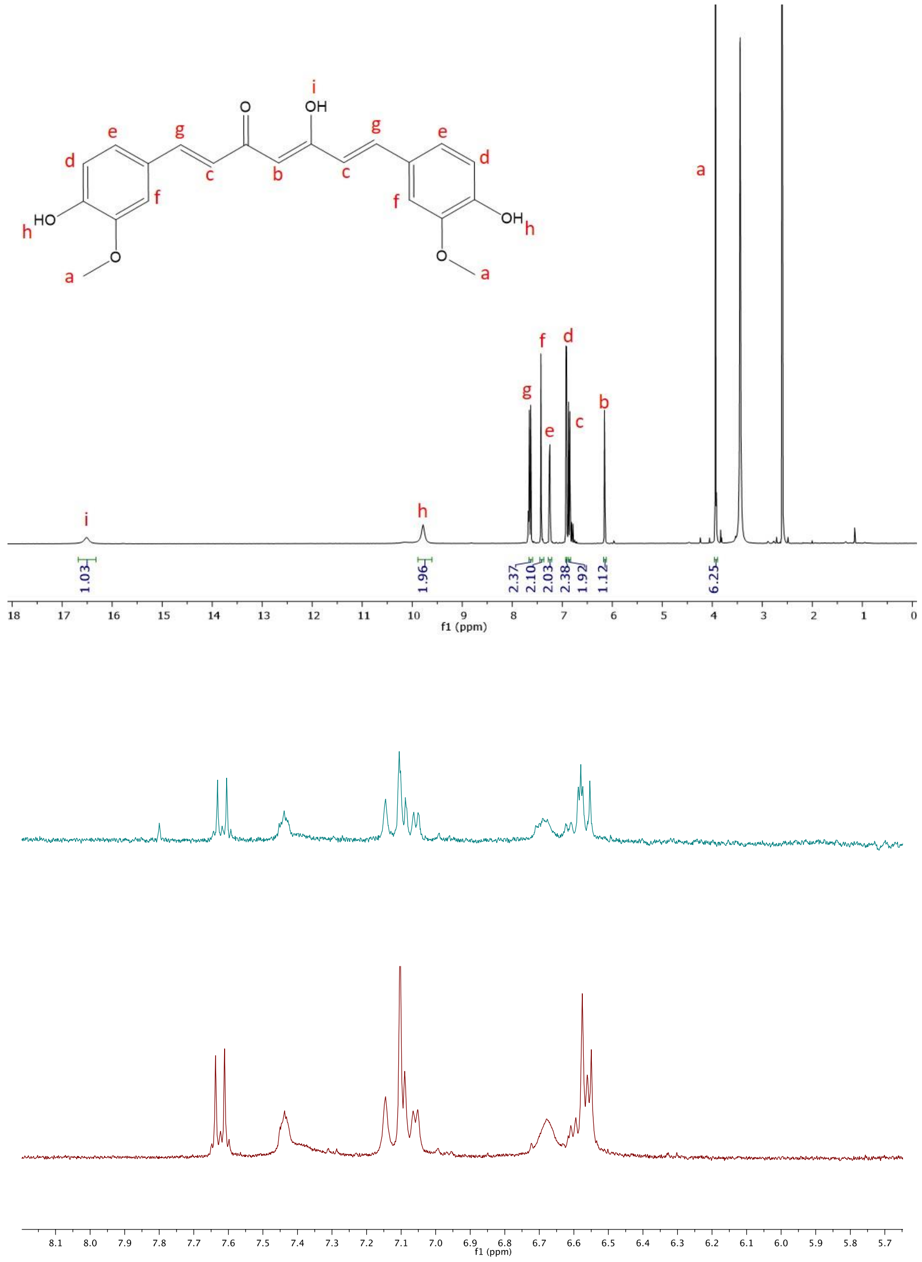

Supplementary Figure 1. top: ^{1}H NMR (600 MHz, DMSO-d_6) spectrum of curcumin DMSO stock solution (2.5 mg/mL)[3] ; bottom: Stacked ^{1}H NMR (600 MHz) spectrum of curcumin (50 μg/mL) in D_2O with (top) and without (bottom) added fructose.

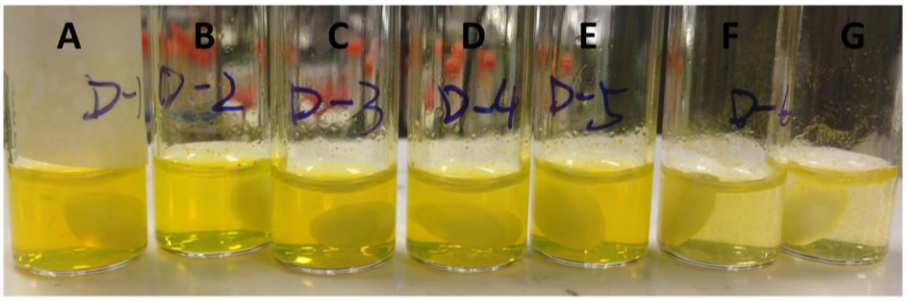

Supplementary Figure 2. Images of curcumin/fructose mixture at different fructose concentration, [CCM] = 50 μg/mL (A) [Fru] = 7.8125 mg/mL (B) [Fru] = 15.625 mg/mL (C) [Fru] = 31.25 mg/mL, (D) [Fru] = 62.5 mg/mL (E) [Fru] = 125 mg/mL (F) [Fru] = 250 mg/mL, (G) [Fru] = 500 mg/mL.

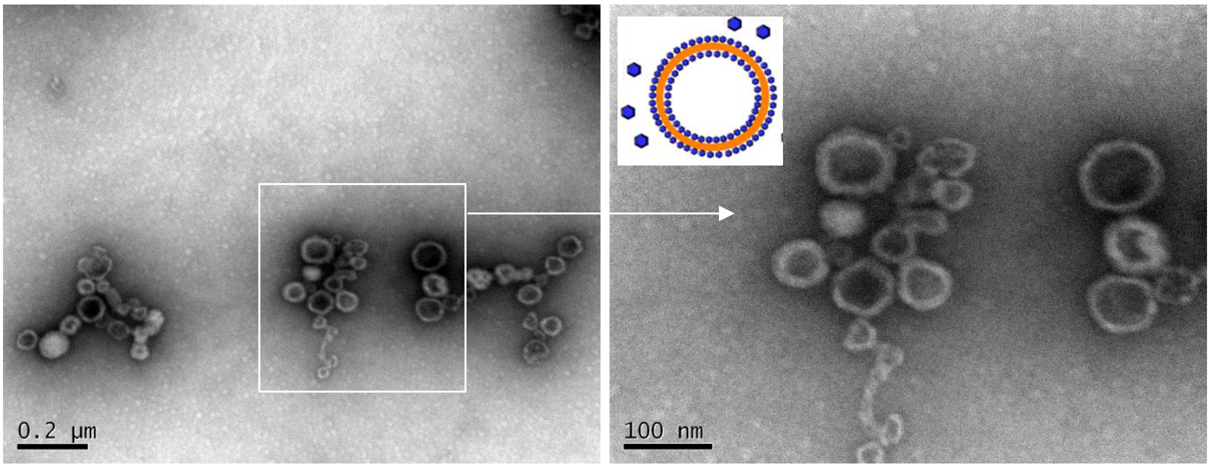

Supplementary Figure 3. TEM images of fructose-curcumin hollow particles. [Fructose] = 10 mg/mL, [Curcumin] = 60 μg/mL, negatively stained with uranyl acetate

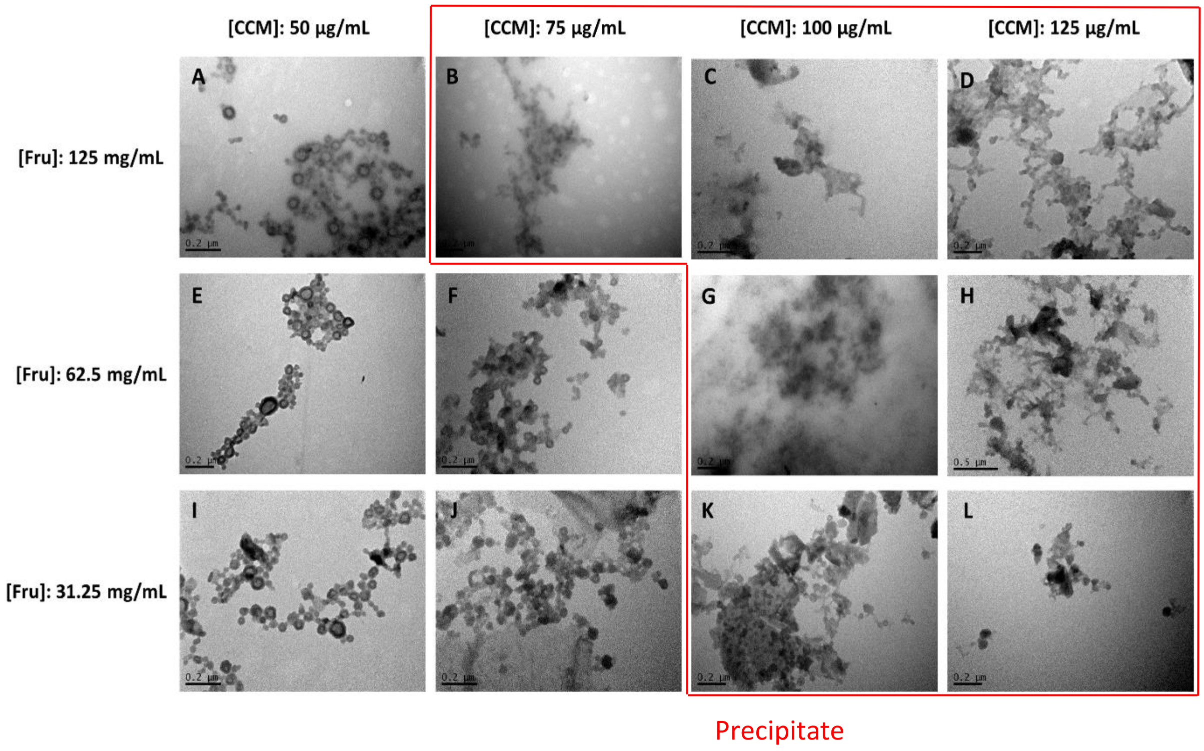

Supplementary Figure 4. TEM images of PDA-Fru-CCM hollow nanoparticles prepared at different curcumin concentration, (A) [Fru] = 125 mg/mL, [CCM] = 50 μg/mL; (B) [Fru] = 125 mg/mL, [CCM] = 75 μg/mL; (C) [Fru] = 125 mg/mL, [CCM] = 100 μg/mL; (D) [Fru] = 125 mg/mL, [CCM] = 125 μg/mL; (E) [Fru] = 62.5 mg/mL, [CCM] = 50 μg/mL; (F) [Fru] = 62.5 mg/mL, [CCM] = 75 μg/mL; (G) [Fru] = 62.5 mg/mL, [CCM] = 100 μg/mL; (H) [Fru] = 62.5 mg/mL, [CCM] = 125 μg/mL; (I) [Fru] = 31.25 mg/mL, [CCM] = 50 μg/mL; (J) [Fru] = 31.25 mg/mL, [CCM] = 75 μg/mL; (K) [Fru] = 31.25 mg/mL, [CCM] = 100 μg/mL; (L) [Fru] = 31.25 mg/mL, [CCM] = 125 μg/mL.

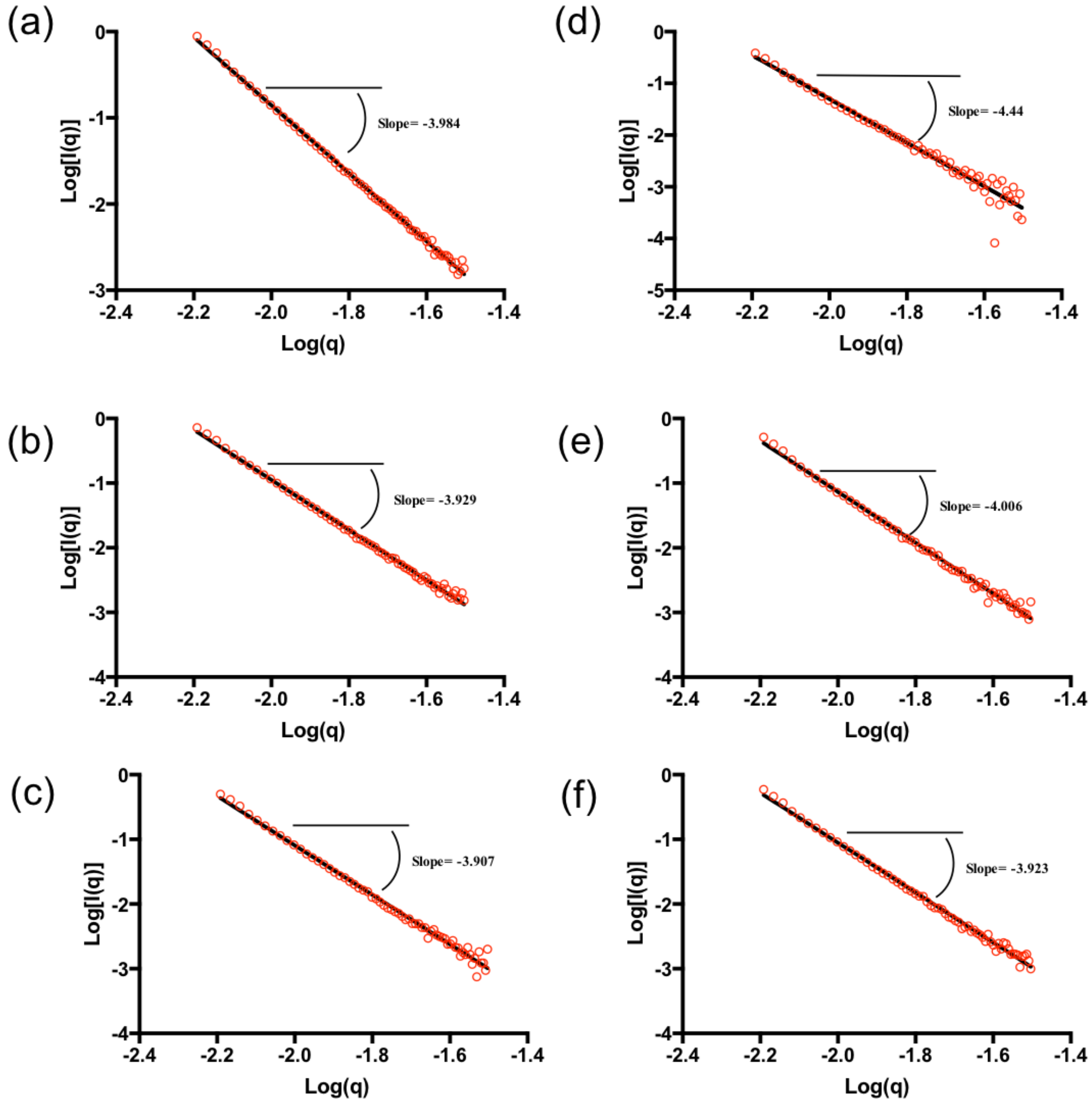

Supplementary Figure 5. (A-C) SAXS data at constant curcumin concentration (10 μg/mL) and various fructose concentration (A, 31 mg/mL; B, 62 mg/mL; C, 125 mg/mL); (D-F) SAXS data at constant fructose concentration (125 mg/mL) and various curcumin concentration (D, 20 μg/mL; E, 60 μg/mL; F, 80 μg/mL).

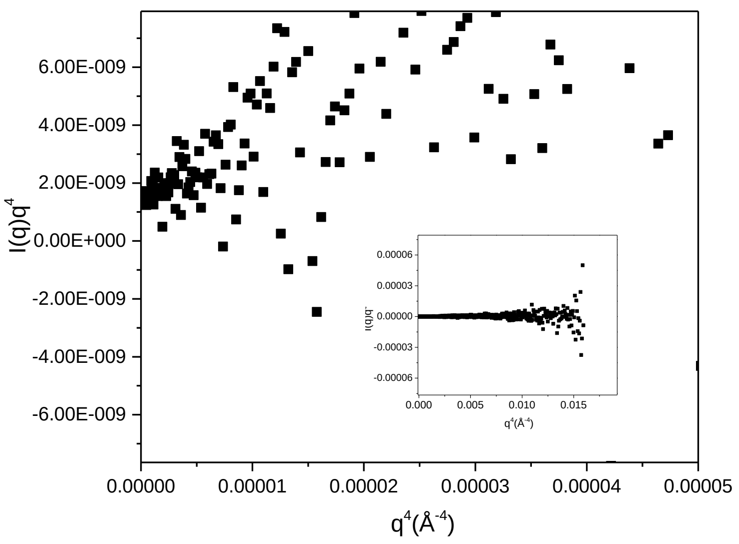

Supplementary Figure 6. Porod plot, $I(q)q^4$ versus q^4 for nanoparticles prepared using [curcumin] = 25 μg/mL and [fructose] = 31 mg/mL). Highlighted region (0.000-0.008 Å^4) was used for extrapolation.

The intercept of the linear fit of the sample shown in Figures S6 is 5.59 × 10^{-10} ± 6.11 × 10^{-12}. The intercept for all samples is within similar range although it seems to decline with increasing curcumin amounts (**Supplementary Figure 7.**).

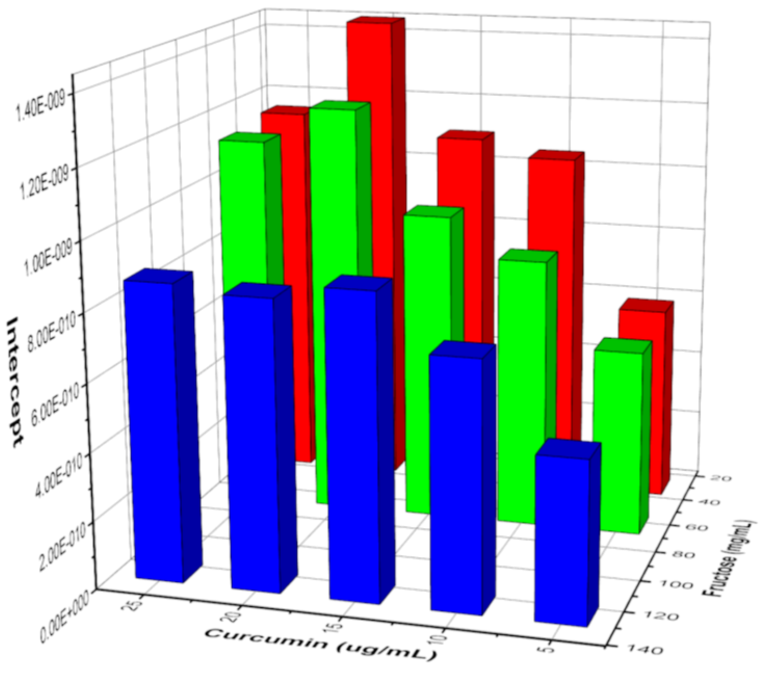

Supplementary Figure 7. The intercept of the intensity plot, $I(q)q^4$ versus q^4 in different amount of curcumin and fructose.

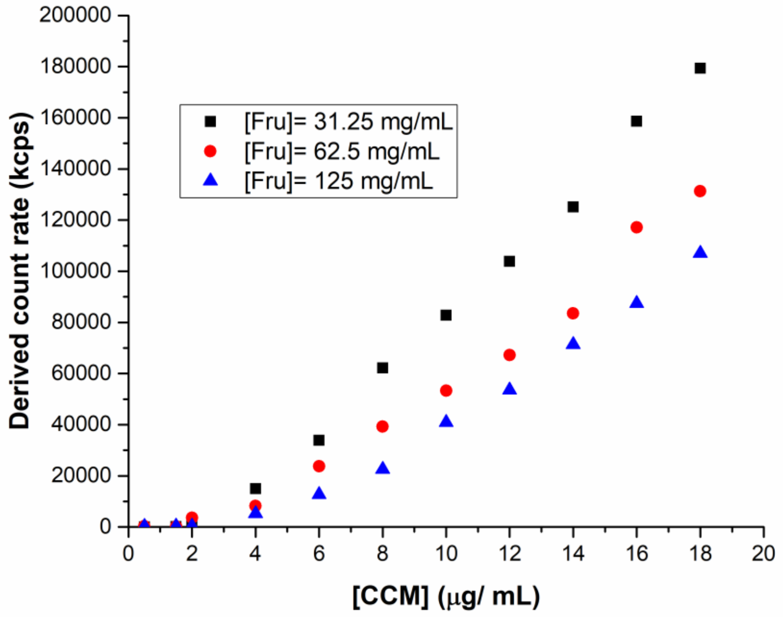

Supplementary Figure 8. Dynamic light scattering (DLS) derived critical aggregate concentration (CAC) calculation.

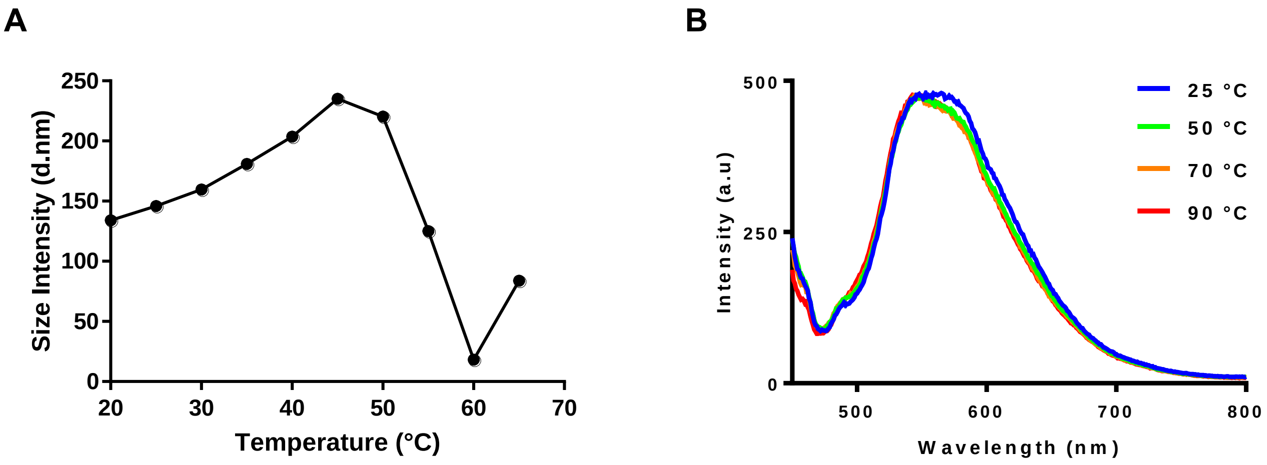

Supplementary Figure 9. (A) Temperature ramp DLS measurements of Fru-CCM nanoparticles from 25 to 65 °C. (B) Fluorescence spectra of curcumin (50 μg/mL, 0.14 mM) and fructose (31.25 mg/mL, 0.17 M) at temperatures of 25, 50, 70 and 90 °C.

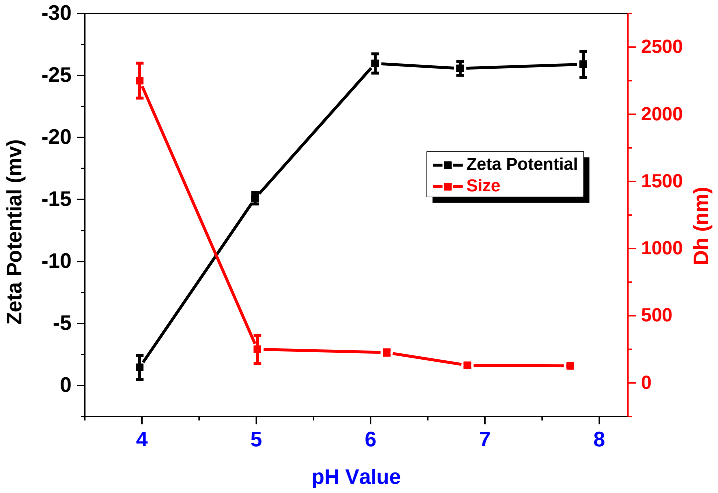

Supplementary Figure 10. Zetapotential measurements and hydrodynamic diameter D_h of the solution of curcumin (50 µg/mL, 0.14 mM) and fructose (31.25 mg/mL, 0.17 M) in dependency of the pH value. The pH from 8 to 4 was controlled by an autotitrator coupled to a Malvern Zetasizer particle size analyser that utilized HCl (0.025 M) and NaOH (0.025 M) additives with stirring.

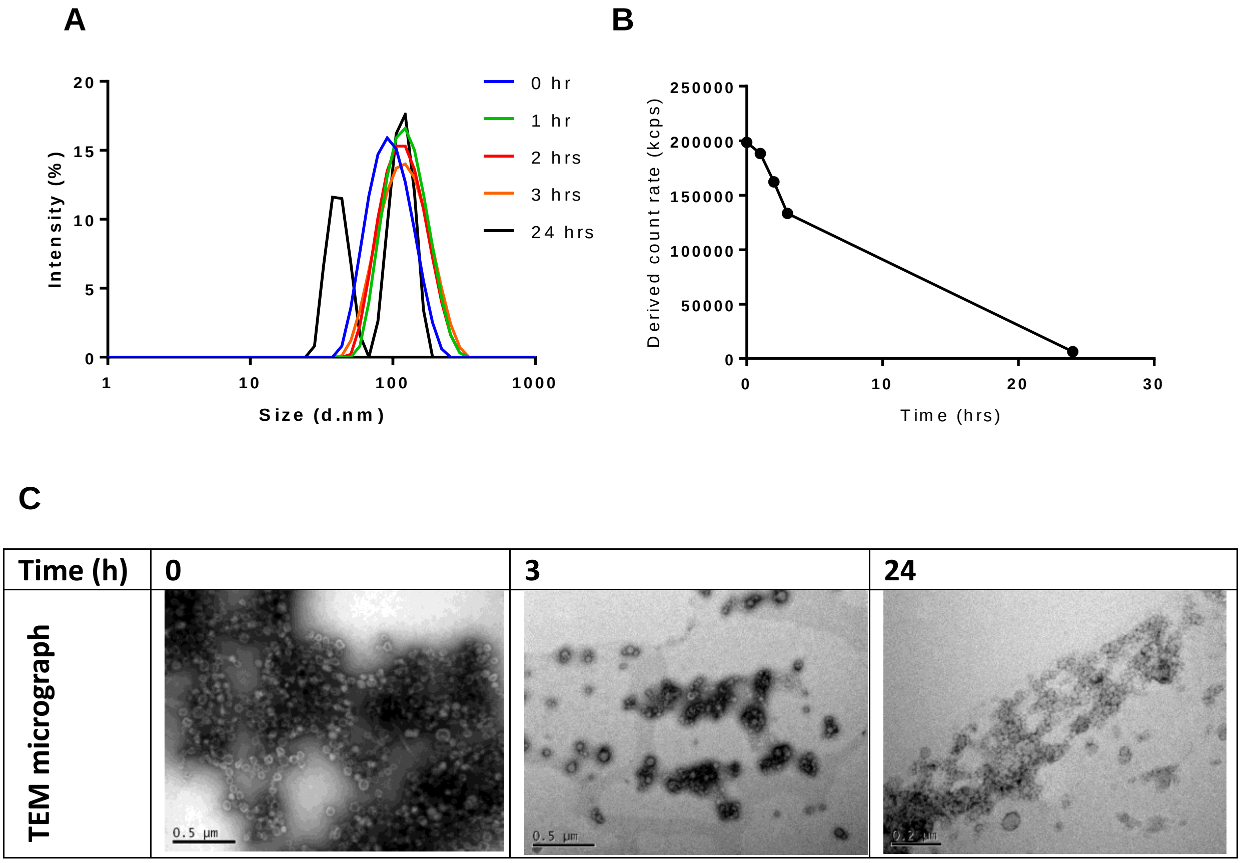

Supplementary Figure 11. [Fru] = 15.6 mg/mL, [CCM] = 50 ug/mL nanoparticles subjected to osmotic pressure (dialysis against 1 L milliQ water) over time represented by: (A) Dynamic light scattering (DLS) intensity size graph and (B) derived count rate of nanoparticles over a period of 24 hours (C) TEM micrographs stained with uranyl acetate at respective time points.

Molecular dynamics (MD) simulations were performed using NAMD 2.12[4] in conjunction with the CHARMM[5] and CGENFF[6] force fields. The CGENFF force field was employed for fructose (β-D-fructopyranose) and curcumin molecules. The CGENFF force field parameters were validated by comparing the calculated interaction energies between a single fructose molecule and a single water molecule at various configurations that were randomly sampled from an NPT trajectory of a fructose molecule immersed in a periodic box of TIP3P waters. The interaction energies were compared with those obtained at the HF/6-31G(d) level (without counterpoise correction using the Gaussian16 program[7]), and the mean absolute deviation is approximately 1.84 kcal mol^{-1} (Table S6). The same comparison was performed for an α-fructofuranose molecule where CHARMM parameters are available, and the mean absolute deviation was very similar around 1.85 kcal mol^{-1} (Table S6). On this basis, we consider the CGENFF parameters for fructose to be satisfactory. For the curcumin molecule, the CGENFF parameters were shown in a previous study[8] to give physically reasonable logP values in good agreement with other theoretical estimates.

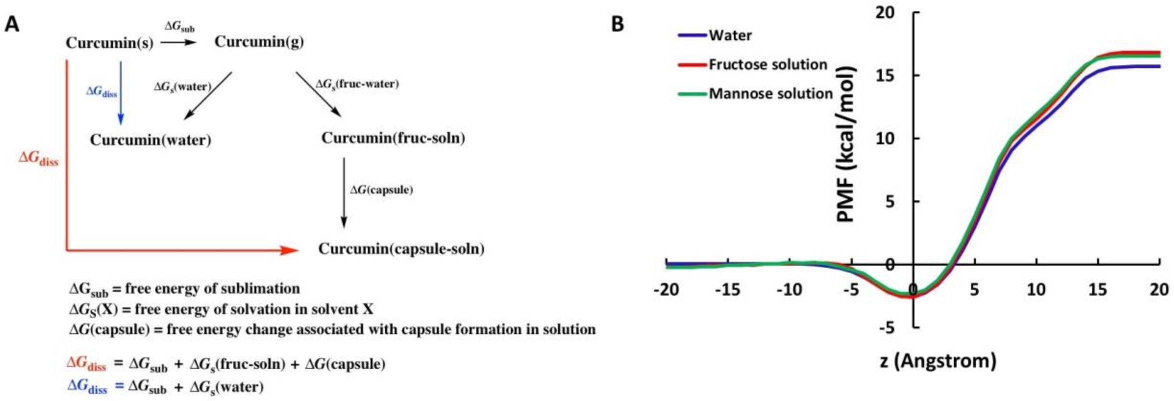

Supplementary Figure 12. (A) A thermodynamic cycle comparing the energetics of curcumin dissolution in water and fructose solution. (B) Potential of mean force as a function of reaction coordinate for the solvation of curcumin in water and fructose solution. A z-value of 0 Å refers to the air-solution interface, and a positive z-value indicates that the solute is above the condensed phase.

Umbrella sampling simulations were carried out to determine the fixed concentration free energy of solvation of curcumin in water an in fructose-water solution. The simulations were performed in a 4 x 4 x 4 nm cubic box of water molecules, using an overall box size of 4 x 4 x 20 nm where the large vacuum region on the end of the simulation cell was used to prevent the solute from interacting with the solvent through periodic boundary conditions. The reaction coordinate for the determination of free energy changes was defined as the distance between the centre of mass of the solute (COMS) under study and centre of mass of the condensed phase (COMCP). The system is oriented such that the reaction coordinate is aligned with the z-axis, and the liquid-vacuum interface lies in the x-y plane. Over the course of simulation, the reaction coordinate spanned a distance of 4 nm from approximately 20 Å deep into the condensed phase. The program Packmol[9] was used to generate the initial configurations for each window (spaced 1 Å apart along the reaction coordinate). To keep the solute within the centre of a specified window, a harmonic force with a magnitude of 2.5 kcal mol^{-1} $Å^{-2}$ was applied. At each window, the system was minimized, and the temperature of the system was gradually increased to 300 K over 1 ns,

before equilibration for a further 2 ns under NPT ensemble (at 1 atm), and a 10 ns production run. The free energy profile (potential of mean force) along the reaction coordinate was obtained using the Weighted Histogram Analysis Method (WHAM) implemented by Grossfield.[10]

To simulate a planar model of the vesicle (8 nm x 8 nm x 12 nm), Packmol was used to generate initial configuration consisting of 200 randomly oriented curcumin molecules, sandwiched between 700 fructose molecules, and water molecules (See Figure 3A left) corresponding to a density of 1 g cm^{-3}. After gradual heating to 300 K under NPT ensemble, the system appears to be stable (zero surface tension was applied in the xy plane; the surface normal is parallel to the z-axis) after 100 ns. The distribution of fructose molecules at a specified distance from the curcumin layer appears to have converged after 40 ns (Figure S13). The 60 ns production run was used to determine the average number of fructose molecules at various distances shown in Figure 3C in the manuscript.

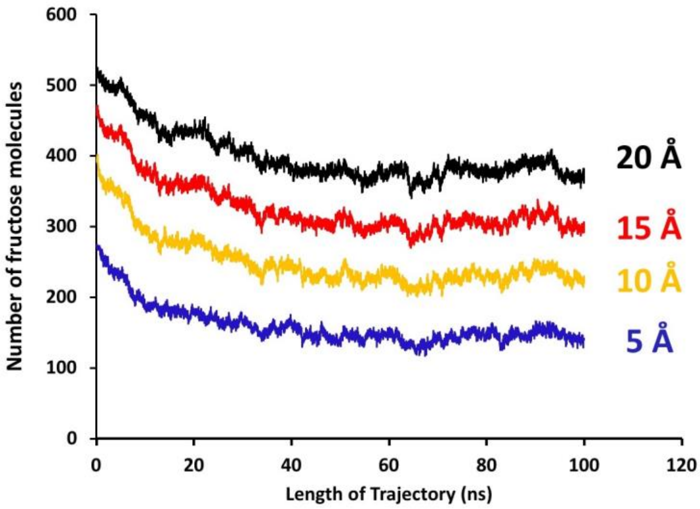

Supplementary Figure 13. The number of fructose molecules at various distances (5, 10, 15 and 20 Å) from the curcumin layer stabilized after approximately 40 ns.

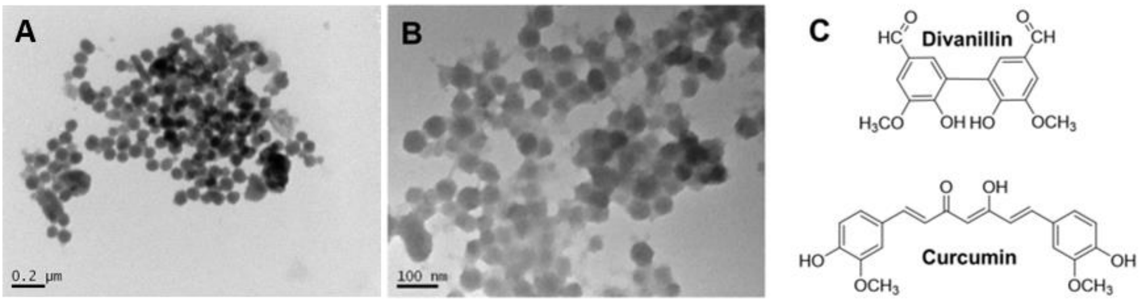

Supplementary Figure 14. TEM images of PDA-Divanillin-CCM nanoparticles prepared at different fructose concentration. (A) [Fru] = 15 mg/mL, [Divanillin] = 10 μg/mL; (B) [Fru] = 61.5 mg/mL, [Divanillin] = 10 μg/mL; (C) chemical structures of curcumin and divanillin.

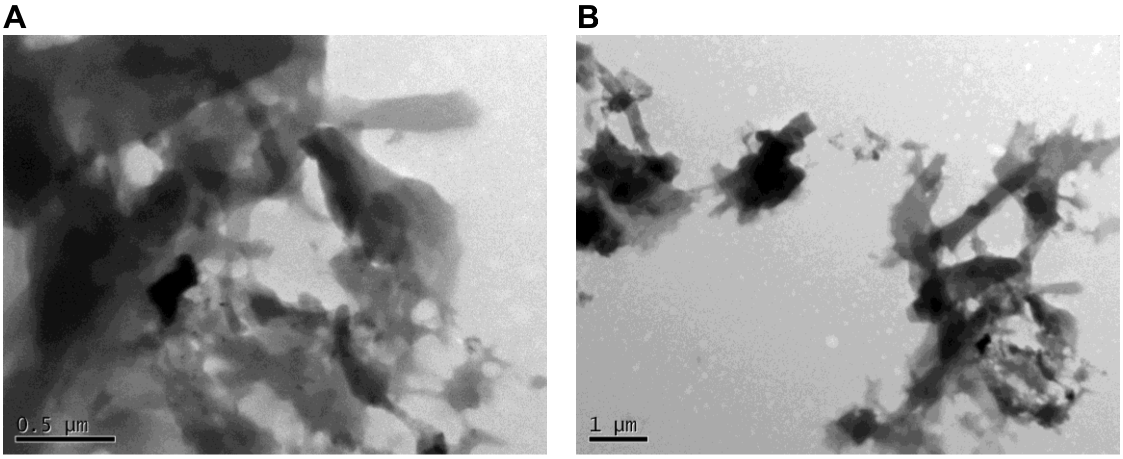

Supplementary Figure 15. TEM micrograph of curcumin aggregates [CCM] = 50 µg/mL in the absence of fructose in aqueous solution (no staining)

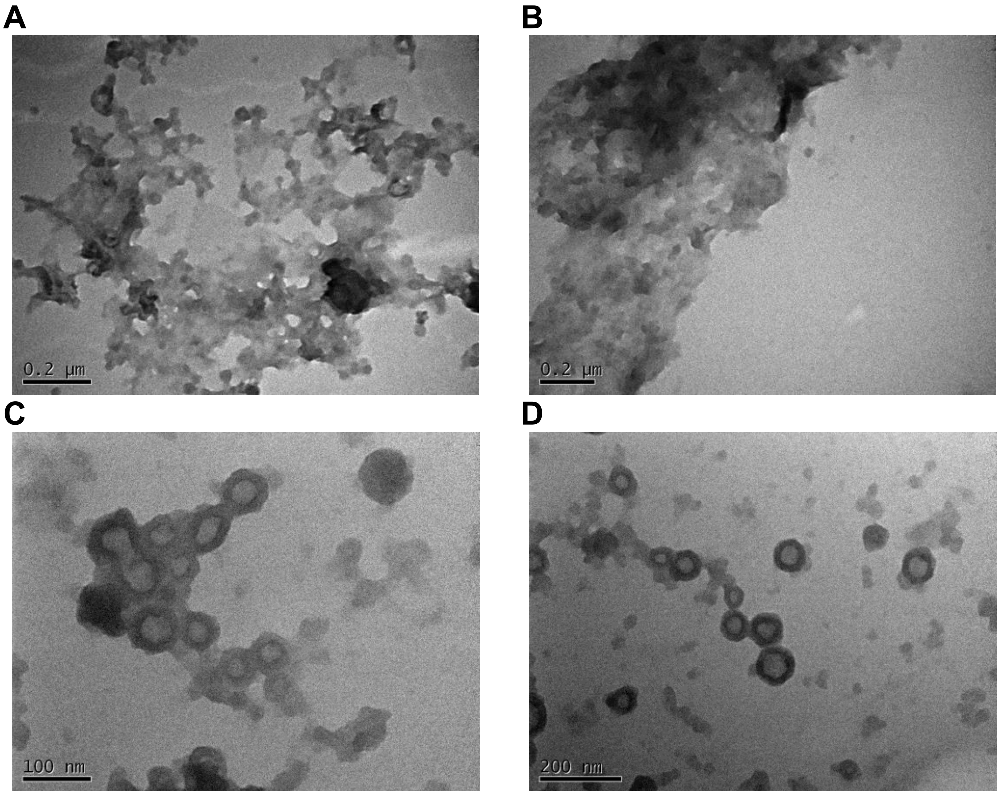

Supplementary Figure 16. TEM images of PDA-Carbohydrate-CCM nanoparticles (A) [Galactose]= 31.25 mg/mL, [Curcumin]= 50 µg/mL (B) [Mannose] = 31.25 mg/mL, [Curcumin] = 50 µg/mL (C,D) [Glucose] = 31.25 mg/mL, [Curcumin] = 50 µg/mL.

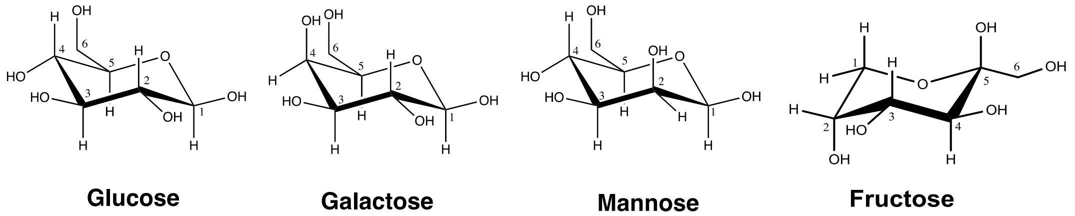

Carbohydrate	OH-1	OH-2	OH-3	OH-4	OH-6
Fructose	-	ax	eq	eq	eq
Glucose	eq	eq	eq	eq	ax
Galactose	eq	eq	eq	ax	ax
Mannose	eq	ax	eq	eq	ax

Supplementary Figure 17. General sugar structure of the six-carbon carbohydrates in the most stable beta-pyranose form, where "ax" refers to axial and "eq" refers to equatorial of the hydroxyl group configuration at carbon positions 2,3,4 and 6.

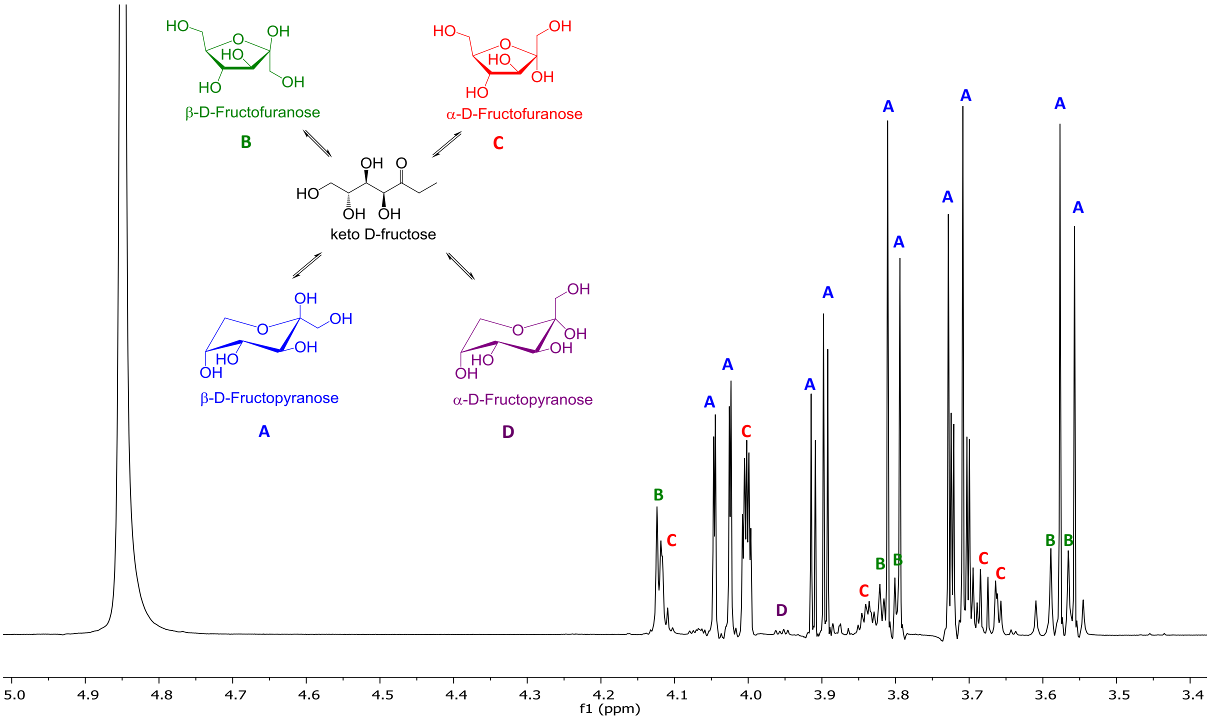

Supplementary Figure 18. ^{1}H NMR spectrum (600 MHz) for D-fructose equilibrated in D_2O (0.17M) at 20 °C.[11]

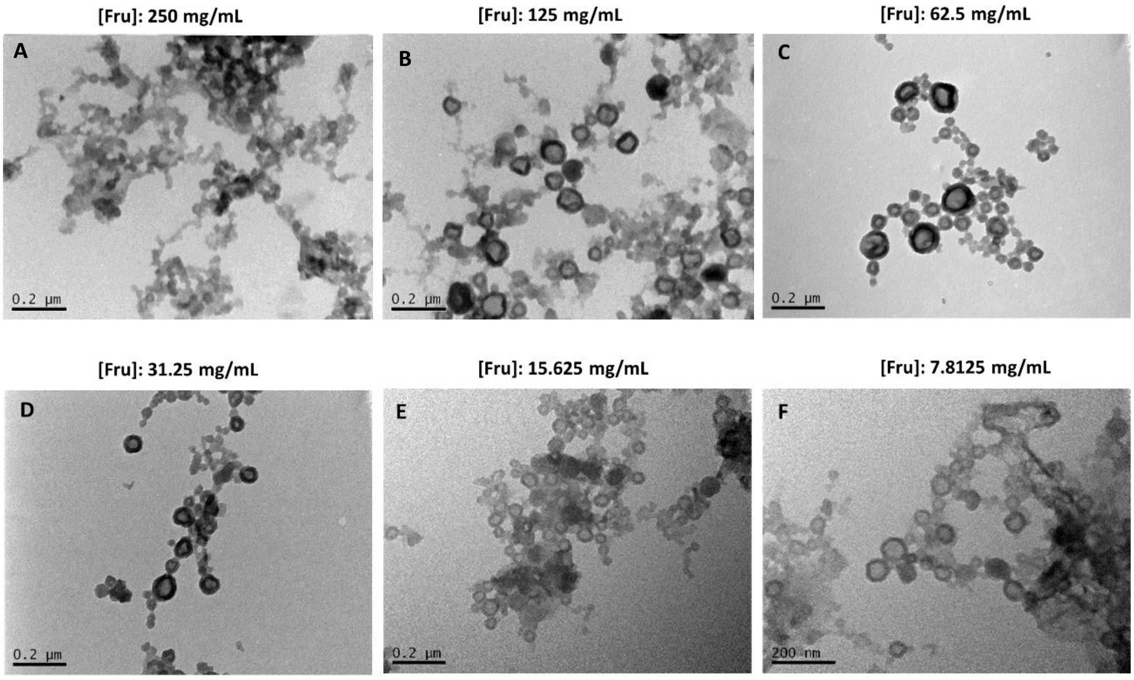

Supplementary Figure 19. TEM images of PDA-Fru-CCM hollow nanoparticles prepared at different fructose concentration, (A) [Fru] = 250 mg/mL, [CCM] = 50 µg/mL; (B) [Fru] = 125 mg/mL, [CCM] = 50 µg/mL; (C) [Fru] = 62.5 mg/mL, [CCM] = 50 µg/mL; (D) [Fru] = 31.25 mg/mL, [CCM] = 50 µg/mL; (E) [Fru] = 15.625 mg/mL, [CCM] = 50 µg/mL; (F) [Fru] = 7.8125 mg/mL, [CCM] = 50 µg/mL.

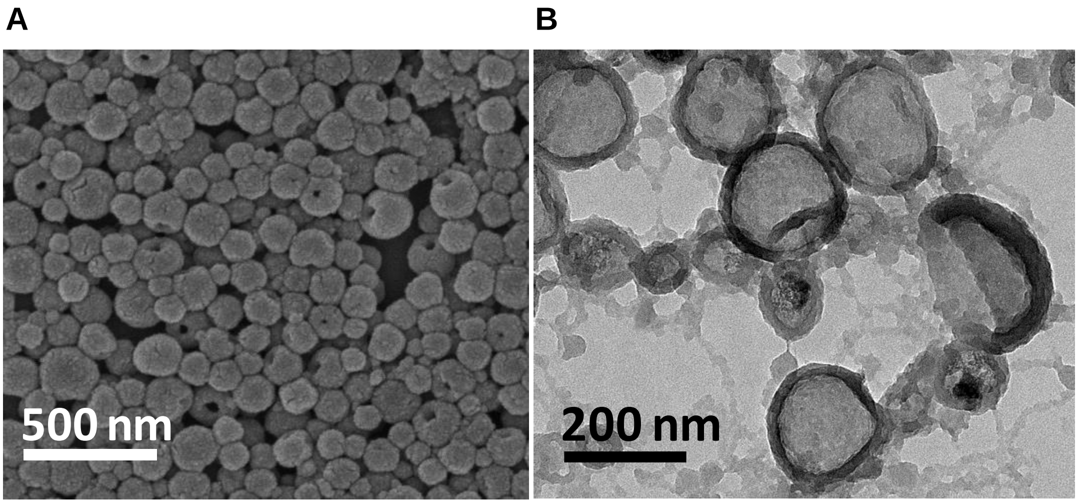

Supplementary Figure 20. PDA-Fru-CCM nanoparticles [Fru] = 10 mg/mL, [CCM] = 60 µg/mL coated with PDA (24 h deposition time). (A) SEM image and (B) TEM image.

Supplementary Table 1. Size of self-assembled capsules from fructose and curcumin.

Sample	[Fructose] (mg/mL)	[Curcumin] (μg/mL)	D_h (nm)	PDI
**1**	50	0	-	-
**2**	50	100	288	0.07
**3**	50	200	296	0.11
**4**	50	300	310	0.16
**5**	10	60	95	0.13
**6**	15.6	50	97	0.027
**7**	31.25	50	120	0.082
**8**	31.25	20	122.1	0.015
**9**	31.25	10	121	0.088
**10**	31.25	5	114	0.056
**11**	62.5	50	122	0.068
**12**	62.5	20	111	0.05
**13**	62.5	10	109	0.074
**14**	62.5	5	103.1	0.053
**15**	125	50	147.7	0.056
**16**	125	20	138	0.072
**17**	125	10	127	0.062
**18**	125	5	112	0.082

Supplementary Table 2. Effect of organic solvent concentration according to DLS measurements

Amount of DMSO (μL)	DMSO (w/w%)	Intensity mean, *d* (nm)	PDI
**40**	2	204.7	0.056
**50**	2.5	209.6	0.052
**60**	3	213.5	0.04
**70**	3.5	226.6	0.104
**80**	4	218.1	0.141
**90**	4.5	177.2	0.496
**100**	5	979.6	0.664

Supplementary Table 3. Size of self-assembled capsules from fructose and curcumin in different solvents

Sample	[Fructose] (mg/mL)	[Curcumin] (μg/mL)	Solvent (40 μL)	D_h (nm)	PDI
**1**	31.25	50	Acetone	123	0.037
**2**	31.25	50	THF	194	0.148
**3**	31.25	50	Ethanol	109	0.138
**4**	31.25	50	DMSO	120	0.082

Supplementary Table 4. The scattering length density (SLD) and volume fractions of fructose, curcumin, H_2O and dimethyl sulfoxide (DMSO).

Compound	Density(g/cm^3)	SLD(Å^{-2})	Volume fraction Ø range in the solution*
**Fructose**[1]	1.69	1.54×10^{-5}	0.018 - 0.074
**Curcumin**[1]	1.3	1.17×10^{-5}	$(1.500 \times 10^{-6}) – (7.700 \times 10^{-6})$
**H_2O**	1	9.47×10^{-6}	0.926 - 0.982
**DMSO**	1.1	1.01×10^{-6}	(2.00×10^{-2}) - (1.00×10^{-2})

* Actual volume fractions are listed;[1] density of fructose and curcumin in the dry state

Supplementary Table 5. The volume fractions of fructose, curcumin, H_2O and DMSO in the solution.

Sample	Fructose (g/mL)	Curcumin (μg/mL)	$Ø_{\mathbf{fructose}}$	$Ø_{water}$	$Ø_{\mathbf{curcumin}}$	$Ø_{\mathbf{DMSO}}$
1	31	5	0.02	0.98	1.54×10^{-6}	2.00×10^{-3}
2	31	10	0.02	0.98	3.08×10^{-6}	4.00×10^{-3}
3	31	15	0.02	0.98	4.62×10^{-6}	6.00×10^{-3}
4	31	20	0.02	0.98	6.15×10^{-6}	8.00×10^{-3}
5	31	25	0.02	0.98	7.69×10^{-6}	1.00×10^{-2}
6	62	5	0.04	0.96	1.54×10^{-6}	2.00×10^{-3}
7	62	10	0.04	0.96	3.08×10^{-6}	4.00×10^{-3}
8	62	15	0.04	0.96	4.62×10^{-6}	6.00×10^{-3}
9	62	20	0.04	0.96	6.15×10^{-6}	8.00×10^{-3}
10	62	25	0.04	0.96	7.69×10^{-6}	1.00×10^{-2}
11	125	5	0.07	0.93	1.54×10^{-6}	2.00×10^{-3}
12	125	10	0.07	0.93	3.08×10^{-6}	4.00×10^{-3}
13	125	15	0.07	0.93	4.62×10^{-6}	6.00×10^{-3}
14	125	20	0.07	0.93	6.15×10^{-6}	8.00×10^{-3}
15	125	25	0.07	0.93	7.69×10^{-6}	1.00×10^{-2}

Supplementary Table 6. Validation of CGENFF parameters for fructose. Interaction energy between fructose and a water molecule at different configurations sampled from MD simulations.

Frame	CGENFF-Fructose	HF/6-31G(d)	$\| \Delta E \|^{a}$	CHARMM-α-furanose	HF/6-31G(d)	$\| \Delta E \|^{a}$
1	-5.29	-5.58	0.29	-4.92	-2.54	2.38
2	-2.83	-2.34	0.49	-3.87	-3.18	0.69
3	-5.58	-1.71	3.87	-7.57	-6.52	1.05
4	-5.92	-5.26	0.66	-4.67	-2.46	2.21
5	-6.42	-5.84	0.58	-5.17	-4.67	0.50
6	-5.63	-2.91	2.72	-9.14	-5.45	3.69
7	-7.15	-5.01	2.14	-3.10	-1.47	1.63
8	-7.61	-5.11	2.50	-4.46	-1.45	3.01
9	-6.92	-5.81	1.11	-0.62	0.58	1.20
10	-2.95	-0.86	2.09	-6.14	-3.19	2.95

11	-4.40	-4.10	0.30	-7.44	-4.53	2.91
12	-5.93	-1.44	4.49	-4.44	-3.37	1.07
13	-5.27	-4.34	0.93	-5.67	-4.75	0.92
14	0.56	2.07	1.51	-5.52	-3.97	1.55
15	-6.51	-5.29	1.22	2.26	4.47	2.21
16	-5.52	-3.07	2.45	-6.72	-4.76	1.96
17	-7.69	-6.20	1.49	-5.80	-4.13	1.67
18	-3.81	-2.66	1.15	-4.09	-3.20	0.89
19	-6.44	-5.09	1.35	-2.27	-0.77	1.50
20	-6.89	-3.39	3.50	-8.64	-6.34	2.30
21	-6.69	-3.73	2.96	-4.94	-3.09	1.85
22	-4.91	-3.70	1.21	-8.45	-4.84	3.61
23	-4.27	-3.04	1.23	-2.19	-1.11	1.08
24	-5.22	-1.37	3.85	-2.97	-1.31	1.66
Mean absolute deviation			1.84			1.85

A The absolute deviation between CGENFF/CHARMM and HF/6-31G(d) (without counterpoise correction) interaction energies.

References

1. Kirby NM*, et al.* A low-background-intensity focusing small-angle X-ray scattering undulator beamline. *J. Appl. Crystallogr.* **46**, 1670-1680 (2013).

2. Christopher J. Garvey IHP, Robert B. Knott and George P. Simon. Small angle scattering in the porod region from hydrated paper sheets at varying humidities. *Holzforschung* **58**, 7 (2004).

3. Payton F, Sandusky P, Alworth WL. NMR study of the solution structure of curcumin. *J. Nat. Prod.* **70**, 143-146 (2007).

4. C. PJ*, et al.* Scalable molecular dynamics with NAMD. *J. Comput. Chem.* **26**, 1781-1802 (2005).

5. Guvench O*, et al.* CHARMM additive all-atom force field for carbohydrate derivatives and its utility in polysaccharide and carbohydrate-protein modeling. *J. Chem. Theory Comput.* **7**, 3162-3180 (2011).

6. Vanommeslaeghe K*, et al.* CHARMM General Force Field (CGenFF): A force field for drug-like molecules compatible with the CHARMM all-atom additive biological force fields. *J. Comput. Chem.* **31**, 671-690 (2010).

7. M. J. Frisch GWT, H. B. Schlegel, G. E. Scuseria, M. A. Robb, J. R. Cheeseman, G. Scalmani, V. Barone, G. A. Petersson, H. Nakatsuji, X. Li, M. Caricato, A. V. Marenich, J. Bloino, B. G. Janesko, R. Gomperts, B. Mennucci, H. P. Hratchian, J. V. Ortiz, A. F. Izmaylov, J. L. Sonnenberg, D. Williams-Young, F. Ding, F. Lipparini, F. Egidi, J. Goings, B. Peng, A. Petrone, T. Henderson, D. Ranasinghe, V. G. Zakrzewski, J. Gao, N. Rega, G. Zheng, W. Liang, M. Hada, M. Ehara, K. Toyota, R. Fukuda, J. Hasegawa, M. Ishida, T. Nakajima, Y. Honda, O. Kitao, H. Nakai, T. Vreven, K. Throssell, J. A. Montgomery, Jr., J. E. Peralta, F. Ogliaro, M. J. Bearpark, J. J. Heyd, E. N. Brothers, K. N. Kudin, V. N. Staroverov, T. A. Keith, R. Kobayashi, J. Normand, K. Raghavachari, A. P. Rendell, J. C. Burant, S. S. Iyengar, J. Tomasi, M. Cossi, J. M. Millam, M. Klene, C. Adamo, R. Cammi, J. W. Ochterski, R. L. Martin, K. Morokuma, O. Farkas, J. B. Foresman, and D. J. Fox, Inc., Wallingford CT. Gaussian 16. (ed^(eds). A.03 edn (2016).

8. Lyu Y, Xiang N, Mondal J, Zhu X, Narsimhan G. Characterization of interactions between curcumin and different types of lipid bilayers by molecular dynamics simulation. *J. Phys. Chem. B* **122**, 2341-2354 (2018).

9. Martínez L, Andrade R, Birgin EG, Martínez JM. PACKMOL: A package for building initial configurations for molecular dynamics simulations. *J. Comput. Chem.* **30**, 2157-2164 (2009).

10. Grossfield A. WHAM: the weighted histogram analysis method. (ed^(eds). 2.0.9 edn.

11. Barclay T, Ginic-Markovic M, Johnston MR, Cooper P, Petrovsky N. Observation of the keto tautomer of D-fructose in D(2)O using (1)H NMR spectroscopy. *Carbohydr. Res.* **347**, 136-141 (2012).
